# Response of Eurasian otters (*Lutra lutra*) to underwater acoustic harassment device sounds

**DOI:** 10.1038/s41598-024-55481-z

**Published:** 2024-02-29

**Authors:** Emilie Nicoline Stepien, Anders Galatius, Kirstin Anderson Hansen, Jacob Nabe-Nielsen, Jonas Teilmann, Magnus Wahlberg

**Affiliations:** 1 Marine Mammal Research, Department of Ecoscience, Aarhus University, Aarhus, Denmark; 2 Marine Biological Research Centre, Department of Biology, University of Southern Denmark, Odense, Denmark

**Keywords:** AHD, Seal scarer, Behavioural response, Habitat exclusion, Feeding disruption, Fishery, Fish farm, Behavioural methods, Experimental organisms, Animal migration, Behavioural ecology, Biodiversity, Conservation biology, Environmental economics, Freshwater ecology, Biological techniques, Ecology, Neuroscience, Auditory system, Feeding behaviour, Learning and memory, Stress and resilience, Zoology, Animal behaviour, Ecology, Animal migration, Behavioural ecology, Biodiversity, Conservation biology, Environmental economics, Freshwater ecology, Macroecology, Environmental sciences, Environmental impact

## Abstract

Seal scarers (or acoustic harassment devices, AHDs) are designed to deter seals from fishing gear and aquaculture operations, as well as to prevent seals from entering rivers to avoid predation on valuable fish. Our study investigated the potential effects of AHDs on non-target species, specifically the Eurasian otters (*Lutra lutra*), by testing the reaction of two rehabilitated otters to simulated AHDs sounds at 1 and 14 kHz, with a received sound intensity of 105–145 dB re 1 µPa rms. The 1 kHz sounds were used to investigate alternative frequencies for scaring seals without scaring otters. The otters reacted to both 1 and 14 kHz tonal signals when retrieving fish from a feeding station 0.8 m below the surface. Their diving behaviour and time to extract food progressively increased as sound intensity increased for all tested sound levels. Notably, the sound levels used in our tests were significantly lower (40–80 dB) than the source levels from commercial AHDs. These findings highlight the importance of caution when using AHDs in river and sea habitats inhabited by otters, as AHDs can change their behaviour and potentially result in habitat exclusion.

## Introduction

The delicate balance between conservation of top predators and economic and recreational exploitation of natural resources often presents complex challenges^1–3^. Seals are an excellent example in the marine environment, as they are both ambassadors for a clean marine environment and a nuisance for fish farmers and fisheries^4,5^. Seals are branded as a tourist attraction in many areas, while in other areas permits are issued to hunt seals or individual seals are regulated close to fish farms and fishing gear^6,7^.

While conflicts between top predators and economic and recreational exploitation of natural resources may arise, recent empirical evidence from sea otter population (*Enhydra lutris*) recovery suggests that these conflicts are not universally present. For instance, a study by Boustany et al.^8^ examined the potential conflict between sea otter recovery and Dungeness crab fisheries in California, revealing that the sea otter population growth and range expansion did not have a negative effect on landings in the crab fishery.

Active management interventions have been instrumental in recovery and reintroduction of Eurasian otters (*Lutra lutra*) in Europe and the North American river otter (*Lontra canadensis*) in North America in areas where otters were extinct or reduced significantly due to historic hunting for their fur or conflicts with fish farming^9^. These programmes aim not only to restore depleted populations of endangered species, but also to reestablish their critical ecosystem functions. In the Pacific Ocean off western North America, similar efforts have been made with sea otter. Notably, these recovery and reintroduction initiatives hold promise for the otter species’ viability and ecosystem sustainability^10,11^.

Acoustic harassment devices (AHDs) are designed to keep seals away from fish farms and fishing gear by emitting intense underwater sounds^12–15^. AHDs may also be deployed to prevent seals from entering rivers to prey upon salmonids^2^. Despite their prevalent usage, AHDs also affect behaviour and hearing of non–target species, such as harbour porpoises (*Phocoena phocoena*) and killer whales (*Orcinus orca*)^16–20^.

AHDs also have the potential of affecting other species. One concern is the Eurasian otter, which has aerial and perhaps also underwater hearing abilities similar to those of seals^21,22^. Otters are usually found in rivers, streams, lakes, and coastal seas. In Denmark, the Eurasian otter was hunted until 1967, but it was still possible to issue hunting permits for otter regulation close to pond farming until 1983^9,23^. Today it is listed as vulnerable on the Danish red list and is rare or extinct in many European countries^24–26^. According to the EU Habitat Directive^27^, deliberate disturbance of otters is prohibited, and activities that may compromise their conservation status should be avoided.

Whereas most AHDs operate at frequencies above 10 kHz, harbour seal behaviour has also been shown to be affected by signals centred at 1 kHz^28^. If AHDs could operate efficiently at lower frequencies, this would most likely reduce impact on non-target species, such as otters, that have a hearing sensitivity in air tapering off below 4 kHz^21^. As there are no studies on underwater hearing of Eurasian otters, it is therefore interesting to measure the response of otters not only to currently used AHDs, but also to signals of lower frequency emphasis.

Here we investigated if Eurasian otters under human care responded to underwater tonal signals with a frequency of 1 and 14 kHz. The sounds were played back at various sound levels to two diving and foraging otters in an enclosure. We examined whether the otters’ response to the sounds were influenced by sound level and frequency, and if there were any indications of individual differences between the two animals or emerging habituation in their reactions.

## Methods

**Figure 1:**
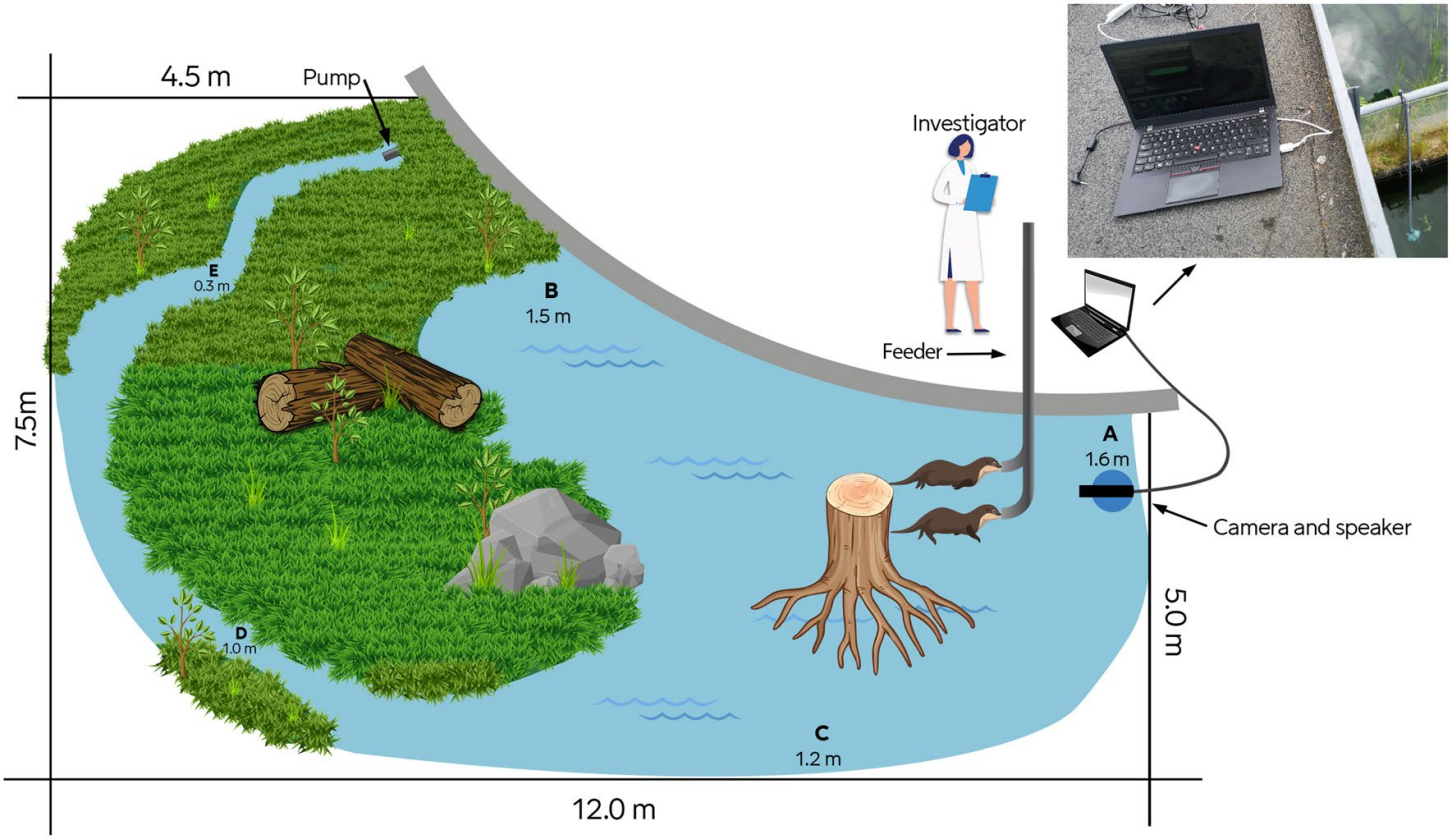
Drawing of otter enclosure in AQUA Aquarium and Wildlife Park, Denmark, illustrating land, logs, water (blue), the two otters at the end of the feeder and the setup in Area A. The letters refer to the areas with various water depths where background noise was measured. Top right: the setup on the roof above Area A, with the laptop connected to the blue underwater speaker visible in the water. The underwater camera mounted above the speaker.

Experiments were carried out at AQUA Aquarium and Wildlife Park (Silkeborg, Denmark) over 11 days during May–August 2019. Two adult otters were included based on availability: a 12-year-old male and a 5-year-old female, both brought into the facility as abandoned pups. Their weight during the experiments is unknown. Both otters were accustomed to aerial noise from the park’s guests and to underwater noise from the water circulation pump of their enclosure. The freshwater pool was irregularly shaped with a depth of 0.3–1.6 m, with concrete walls and a bottom covered by sand and rocks (Fig. 1). Due to its irregular shape, the pool was not notably reverberant during playback. The water temperature was 15–22 °C. The underwater background noise level was measured regularly at different locations and various depths (A–E, Fig. 1) using a SoundTrap ST202HF (Ocean Instruments, Inc.; sampling rate 48 kHz; 16 bits; preamplifier gain high; clipping level 176 dB re 1 µPa p, determined by relative calibration^29^. The spectral density of background noise and playback signals were measured in Matlab (Mathworks, Inc, version 2019b) using Welch’s method^29^ (FFT size 2048 points, 50% overlap and Hann windowing).

Two tonal signals at different frequencies were played back: the 1 kHz signal contained several harmonics, some of which were as intense as the fundamental, whereas for the 14 kHz the harmonics had an intensity more than 40 dB below the fundamental (see supplementary material). Both signals lasted 500 ms, including 100 ms ramp-up and -down. Five stimulus levels were played back: 105, 115, 125, 135, and 145 dB re 1 μPa rms@1 m (measured in a 95% energy level window; see^30^ for details). Playback signals were measured 1 m from the speaker using the same SoundTrap as described above, with a maximum received level variation of ± 6.9 dB around the otter’s location at the feeder.

The signals were played from a WAV file (sampling rate 48 kHz, 16 bits) in randomized sound pressure level order in Area A (Fig. 1) from an Electro-Voice UW-30W loudspeaker and a Sonar Product HS25 spherical transducer, attached to a vertical PVC pipe at a depth of 0.8 m and connected to a laptop computer with Adobe Audition (Adobe, Inc.). An underwater video camera (Divers pro fish-eye 10-021, LH-camera, Fredericia, Denmark) was mounted inside a PVC pipe just above the speaker facing the otters. The camera was connected via an Elgato video capture USB device (Elgato, Corsair Gaiming, Inc., Germany) to a laptop computer, which recorded both video and emitted acoustic signals (Fig. 1).

One or two playback sessions (at 9 am and/or 1 pm) were made every experimental day, with 1–61 days between experimental days. During each experimental session, up to 7 sounds were played according to a randomized list mixing frequency and sound level. Up to 5 control trials were also randomly inserted into the trial list for each session. Control trials were identical to exposure trials but without any sound being played back. Experimental sessions were performed outside the otters’ normal feeding sessions (at 11 am and 2 pm), using 200–300 g fish of their daily ration (800 g for the female and 900–1000 g for the male) for each experimental session. To minimise the risk of habituation, in each experimental session there were never more than 3 consecutive trials in a row with the same signal, and never more than 3 consecutive control trials. During the entire experimental period, the otters were exposed to each sound level 10–18 times for each frequency, with up to 10 exposures per otter for each frequency and sound level. The signals were played back at random intervals, with at least 1 min. between trials.

The experimenter was located on the roof of the building approximately 5 m above Area A (Fig. 1) to minimise visual distraction of the otters. Before each session, a feeder with two netted tubes containing 2–4 pieces of common roach (*Rutilus rutilus*) were lowered from the roof to a depth of 0.8 m, at a distance of 1 m from the speaker. The feeder was first introduced without any sound present for 2 days. Experimental sessions began once the otters associated the feeder with food. The experimenter manually controlled the transmission of the signals, the video recording, and lowering and filling of the feeder, while observing the otters on a laptop via the underwater camera.

The experiments were made under a permit from the Danish Animal Experiments Inspectorate (Ministry of Food, Agriculture and Fisheries of Denmark, permit nr. 2023-15-0201-01608). The experiment was within the frame of the permission to keep the rehabilitated animals under human care. The study is reported in accordance with ARRIVE guidelines (www.arriveguidelines.org).

### Recording animal behaviour

**Figure 2:**
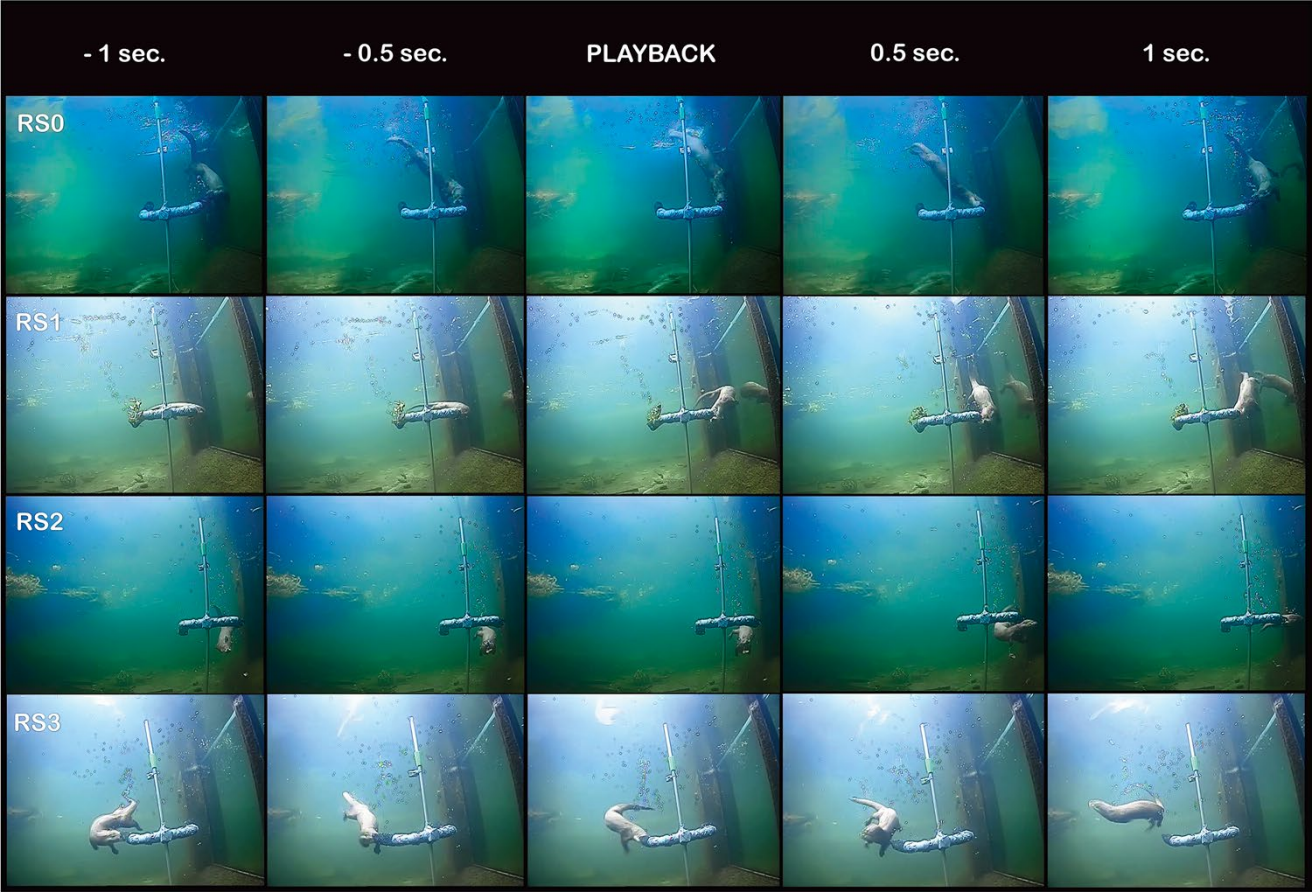
Video frame sequences of a trial with no response (RS0) for 14 kHz at 115 dB re 1 µPa, minor response (RS1) for 14 kHz at 115 dB re 1 µPa clear response (RS2) for 14 kHz at 125 dB re 1 µPa, and a startle response (RS3) to 14 kHz at 135 dB re 1 µPa. The sequences show the otter 1 s before until 1 s after playback. The feeder was placed at 0.8 m depth facing the speaker at a distance of 1 m.

All trials started at a randomized time (range 16 s–5:28 min) after the feeding station was in place in front of the speaker and underwater camera, to prevent the otters from predicting the timing of the signal. Playback was first initiated when the otter’s head faced the feeder in an attempt to take a piece of fish. Data were collected separately for each individual otter present at the feeder during each trial via the video recordings. Behavioural responses were graded by two independent observers watching the recorded videos and using Response Scores (RS) from 0 to 3 (defined in Table 1, with examples given in Fig. 2). A behavioural response was defined by the otter orienting its head and/or body away from the feeder from playback start until 1 s after playback (Fig. 2). The scores of the two observers were not significantly different (Kruskal–Wallis test, df = 1, *n* = 191, *p* = 0.6), so the average observer score was used in the analysis. A response was defined as any score above a non-response, which was defined as no change in head and/or body position and denoted RS0. Control trials followed the same analysis protocol as stimulus trials.

**Table 1:**
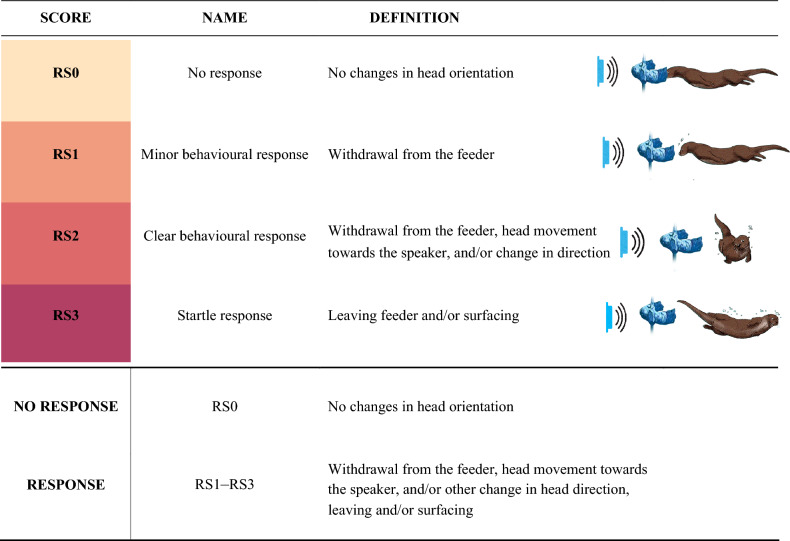
Definitions of response scores RS0–RS3.

RS1–RS3 were used for RESPONSE, and RS0 for NO RESPONSE when defining the binary variables generalized linear model.

Dive duration was measured from the moment the otter's head submerged beneath the water until it resurfaced. If the otter went out of the field of view before resurfacing was observed, the dive duration for that instance was not recorded. The number of dives were counted for each dive performed, from the first submersion until a fish was retrieved and the animal returned to the surface. It included dives where no fish was retrieved. The duration of fish extraction from the feeder was measured from the moment the otter first interacted with the mouth of the feeder until the second a fish was successfully extracted, including time they spent at the surface after unsuccessful attempts and did not include the time spent consuming the fish. Otters never swallow fish under water^31^ but bring them to the surface to consume them. After eating a piece of fish, the otter returned to the feeder, and a new recording of dive duration, number of dives and time to get the fish began. The session stopped when the feeder was emptied. All data points were included in the analysis.

The study aimed to investigate the behavioural response, dive duration, number of dives, and the time to get fish out of the feeder, and how each of these response variables were affected by sound level, frequency, animal ID, number of trials, and exposure. The following statistical models were used to analyse the data in R (*v*. 3.5.2, 2018): for the behavioural response (binary: response/no response), a logistic regression model was fitted using the generalised linear model, *glm* function with logit link. To analyse dive duration (time in seconds), number of dives and the time to extract fish, normality was assessed using the Shapiro–Wilk test from the *stats* package^32^. As the data were not normally distributed, a gamma regression model was fitted using the *glm* function with frequency, sound level, animal ID, trial number, and the interaction between animal ID and the before mentioned predictors. Dive duration of otters and the time spent on extracting fish were analysed using a generalized linear model with gamma distribution and log link function. The number of dives was fitted Poisson and negative binomial regression models, and a likelihood ratio test was performed to compare the two models, which showed that the negative binomial model was the best fit (Chisq = 8.5, df = 7, *p* = 0.29). We used stepwise regression for all model selections using the *step* function from the *stats* package^32^. This involves removing one predictor variable at a time until all remaining predictor variables were significant at a level of 0.05. After the final model was selected, we used diagnostic plots to check the assumptions of the model, including normality of residuals and homoscedasticity. All graphs were made using *ggplot2* package in R^33^.

## Result

We performed 18 playback sessions over 11 days, resulting in a total of 57 playback and 49 control trials. Each session lasted from 23 min to 1 h 24 min. The otters used on average 2.1 min. to collect and eat each fish before returning to the feeder, ready for a new trial. In total, there were 10–18 trials for each frequency and intensity combination, with either one or both otters present at the feeder.

During the trials, the underwater ambient noise levels at the site of the feeder varied with less than 5 dB in the frequency range of sound stimulus (1 and 14 kHz, respectively; see supplementary material). When the circulation pump was turned off during sessions and on between sessions, the noise level increased by about 40 dB in the enclosure in the 1–14 kHz frequency range when the pump was turned on (see supplementary material).

The behavioural responses of both animals were investigated using the binomial logistic regression model (*glm*, binomial, *n* = 190; Table 2). The intensity of acoustic stimuli had a significant impact on the behavioural response exhibited by the otters (*p* < 0.001). The odds ratio for sound level can be calculated as exp(0.028) = 1.03, which indicates that for a one–unit increase in sound level (dB), the odds of observing a behavioural response increase by a factor of 1.03, holding all other predictor variables constant. The difference in individual response was not statistically significant (*p* = 0.06). The binomial regression model had the lowest AIC value = 178.2, and the model’s goodness of fit was measured using the null and residual deviance values (264.8 and 172.2, respectively). They indicate how well the model fits the data. A lower deviance value indicates a better fit. In this case, the residual deviance is lower than the null deviance, which suggests that the model explains more of the variation in the data than would be expected by chance. Therefore, we can conclude that the model is statistically significant and provides a good fit to the data. The dispersion parameter for the binomial distribution was estimated to be 1. This parameter measures the degree of overdispersion or underdispersion in the data. A value of 1 indicates that the data are neither overdispersed nor underdispersed but are homogeneously distributed. In other words, the behavioural responses of the observed individual otters are consistent with each other.

**Table 2:**
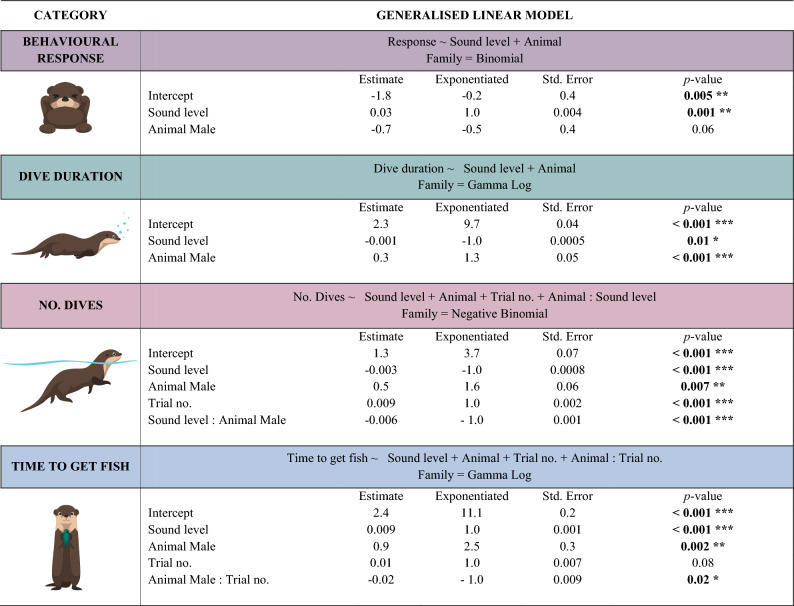
Generalised linear models (*glm*) for two otters’ behavioural responses (binomial) at 0, 1, and 14 kHz, dive duration (gamma log), number of dives (negative binomial), and time spent to get fish out of the feeder (gamma log). Exponentiated is the exponentiated value of the estimate (exp^Estimate^).

Significant *p* value is illustrated with < 0.05*, < 0.01**, < 0.001***.

**Figure 3:**
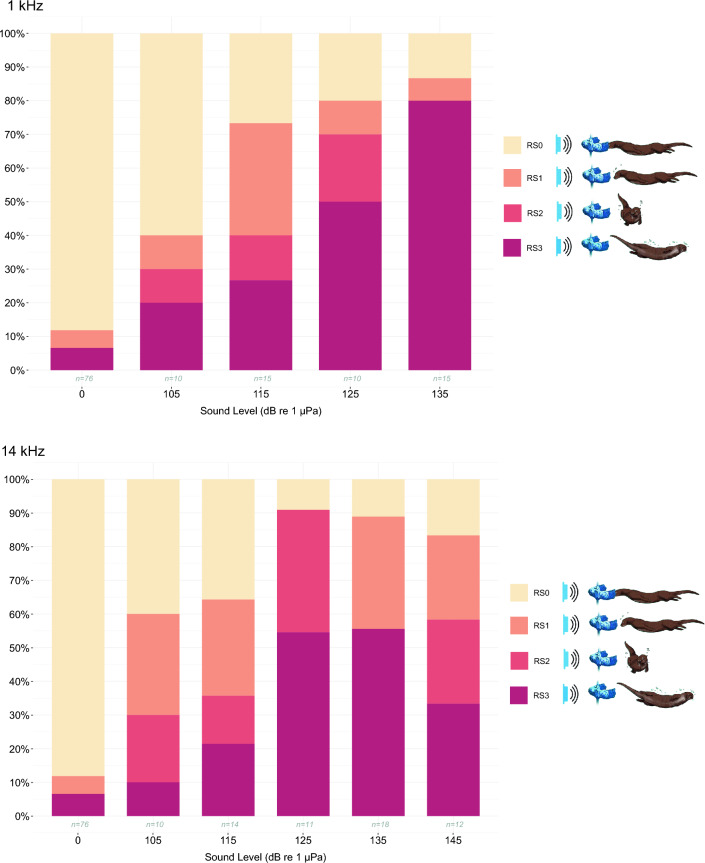
Stacked bar chart for behavioural response scores (RS0–3) for the different stimulus levels and controls, for1 kHz (top) and 14 kHz (bottom) at received levels of 105–145 dB re 1 µPa.

Behavioural response scores increased with increasing received sound level (Fig. 3). For 1 kHz the behavioural responses (RS1–3) increased from 40 to 73% between 105 and 115 dB re 1 μPa, respectively, with RS1 being responsible for 27% of the total response (Fig. 3). The responses then increased to 80–87% at 125–135 dB, with a decrease in RS1 and RS2 and an increase in RS3 at 135 dB re 1 μPa. For 14 kHz the responses increased from 60–64% from 105 to 115 dB re 1 μPa, with 30% of responses ranked as RS1. From 125 to 135 dB re 1 μPa, RS3 accounted for 55% of 90% behavioural responses. There were fewer startle responses (RS3 with33% of 83% behavioural response) at 145 dB compared to 125–135 dB re 1 μPa. RS2s were mostly observed at 125 and 145 dB re 1 μPa (25–36% of the responses). There was an obvious lower response rate during controls (5% for RS1 and 7% for RS3).

According to the generalized linear model with a gamma distribution and log link function, the duration of dives was found to be influenced by sound level and individual *(glm*, gamma log, *n* = 462, *p* < 0.05; Table 2, Fig. 3). The analysis revealed that an increase in sound level was associated with a decrease in dive duration, with each unit increase resulting in a reduction of approximately 1 s (*p* = 0.01). Additionally, the analysis showed a notable effect on individual on dive duration, with the log of the response time expected to increase by 1.6 s for the male otter compared to the female (*p* < 0.001). The negative binomial regression model had the lowest AIC value = 2930.9, and both null and residual deviance values (185.1 and 175.1, respectively) confirmed the statistical significance of the model (*p* < 0.05). The dispersion parameter for the negative binomial distribution was estimated to be 0.3, which indicates that the variance of the duration of dives is proportional to the mean duration of dives.

**Figure 4:**
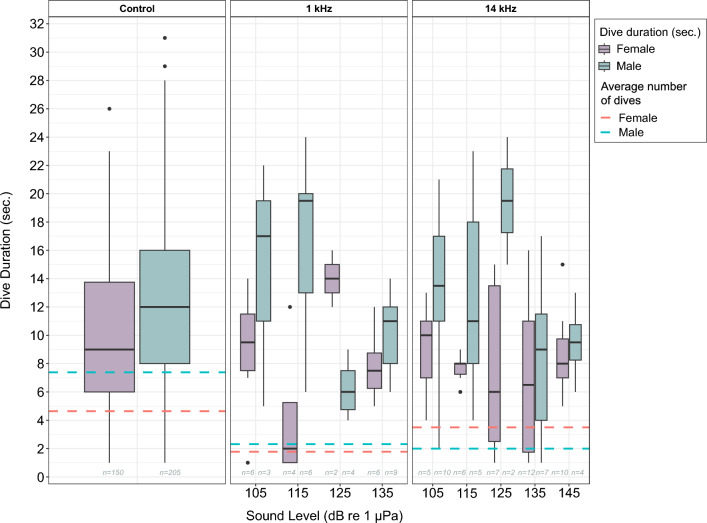
Dive duration (s) with no sound (control, left), 1 kHz (middle) and 14 kHz (right) playback exposures at 105–135 dB re 1 µPa. The average number of dives is shown a stippled lines for the female (pink) and the male (green) otter.

The investigation into the number of dives revealed significant influences from sound and the number of trials, with differences between the two animals (*glm*, negative binomial, *n* = 462, *p* < 0.005; Table 2). Specifically, an increase in sound level was associated with a decrease in the number of dives, with each unit increase in sound level resulting in a reduction of approximately one dive (*p* < 0.001; Table 2; Fig. 4). The number of trials positively correlated with an increase in the number of dives by one (*p* < 0.001). Notably, the male otter exhibited a higher dive frequency in general, recording 1.6 more dives than the female (*p* < 0.001), but reduced his diving frequency by 1.0 more compared to the female with increasing sound level (*p* < 0.001).

The negative binomial regression model had the lowest AIC value = 2255.6, and both null and residual deviance values (658.1 and 448.7, respectively) confirmed the statistical significance of the model (*p* < 0.005). The dispersion parameter for the negative binomial distribution was estimated to be 6.3, underscoring the heterogeneity in dive behaviours between the two otters.

**Figure 5:**
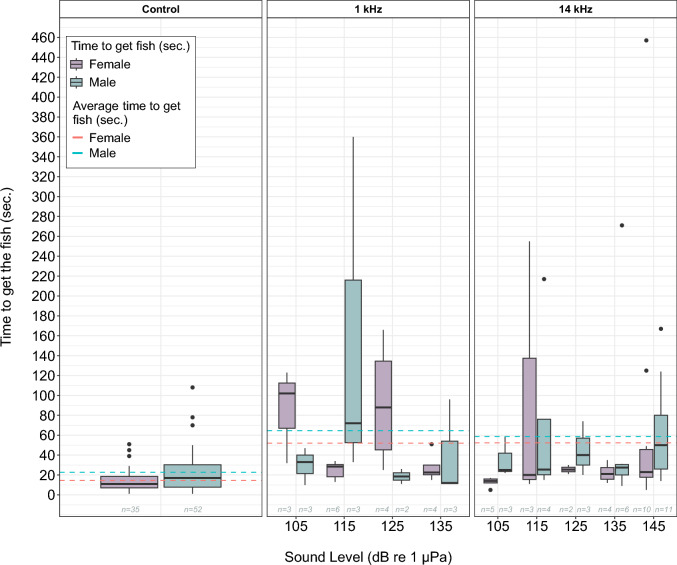
Time (s) spent getting the fish out of the feeder with no sound (left), for 1 kHz (middle) and 14 kHz tones (right) for both female (purple) and male otter (green) divided by the source level 105–145 dB re 1 µPa. The overall average time for each animal to get the fish for the two frequencies is illustrated with the dashed lines (pink for female and green for male).

The time the otters spent extracting a fish from the feeder was significantly affected by sound level, individual and number of trials (*glm*, gamma log, *n* = 165, Fig. 5). The analysis showed that an increase in sound level was associated with an increase in the time to get fish, with each sound level unit increase resulting in an increase of approximately one second (*p* < 0.001). There was a notable effect of individual on the time to get fish, with the log of the response time expected to increase by 2.5 times for the male otter compared to the female (*p* = 0.003). The number of trials significantly reduced the time for the male by one second (*p* = 0.02) while it increased one second for the female, though it was not statistically significant (*p* = 0.08).

The gamma log-linear regression model with the lowest AIC value = 1480.2, had a null deviance of 195.7 and the residual deviance of 140.2 provided a statistically significant model (*p* < 0.05). The model further revealed a dispersion parameter of 1.2, highlighting the variability in the response variable. Overall, these findings underscore the nuanced interplay between sound level, individual characteristics, and trial dynamics in shaping the temporal aspects of otter behaviour when exposed to sound during fish extraction from a feeding station.

## Discussion

This study investigates the underwater auditory capabilities and behavioural response of Eurasian otters to frequencies produced by AHDs. Both the male and female otter exhibited the ability to perceive 1 and 14 kHz sounds underwater, responding distinctly to stimuli of varying intensities. The behavioural responses showed a progressive increase as sound intensity increased from 105 to 145 dB re 1 μPa, with minimal responses during control trials, indicating otters’ sensitivity to presented underwater sounds (Fig. 3).

Our findings suggest that increased sound levels led to a reduction in both the duration and the number of dives by approximately one second and one dive, respectively. Furthermore, the time taken by the otters to extract fish from the underwater feeder increased by one second per elevated sound level, indicating a disruptive effect on otters’ foraging behaviour, making them spend more time at the surface.

Individual differences played a significant role, with the male otter exhibiting 1.3 s longer dive duration and a higher baseline dive frequency, recording 1.6 more dives than the female. Interestingly, the male otter reduced his diving frequency by one dive more compared to the female with increasing sound levels, suggesting a nuanced interplay between individual, sound exposure, and foraging behaviour. The male otter also took 2.5 times longer than the female to extract fish, but as trial numbers increased, the extraction time decreased. This could imply potential differences in caution or the ease of extracting fish between the two individuals, where the male was initially more cautious or faced greater challenges in extracting fish. The observed reduction in extraction time over trials suggests that the male adapted more quickly to the experimental setup compared to the female. This indicates potential adaptability differences between the individuals within the experimental setup.

The experiments were conducted in shallow water, and the otters only had to dive to 0.8 m to reach the feeding station. Nolet et al*.*^34^ suggested that otters tune their dive times to an expected success rate at the corresponding depth. The increase in the number of dives with the number of trials may be attributed to the animals adapting their diving behaviour as they learned how to retrieve fish from the feeder, ultimately reducing the time needed to extract fish. The reduction in the number of dives could likely be due to annoyance caused by playback sounds, emphasising the complex interplay between acoustic stimuli and otter behaviour. The experimental animals were raised by humans and exposed to high levels of noise from visitors in the public aquarium and water pumps. The minor behavioural responses observed at the lowest received sound levels may be explained by the sounds being less annoying due to other acoustic disturbances. Also, the otters may have been highly motivated to stay near the feeder for obtaining food, overruling any urge to move away from the sound.

These results collectively underscore the multifaceted nature of factors influencing diving behaviour, shedding light on the interplay between acoustic stimuli, trial dynamics, and inherent individual differences in otter behaviour. Overall, our study provides new insights into the effects of sound exposure on otters’ behaviour and highlights the importance of considering individual differences and trial number when analysing otter behaviour.

As both otters responded at all sound levels played here, we conclude that the otters’ response threshold was below 105 dB re 1 µPa both at 1 and 14 kHz. There were a few responses in the control experiments, which could be caused by other disturbances from e.g., staff, visitors, or other anthropogenic noises. The observed head movements in the clear behavioural responses (RS2) were always directed towards the underwater loudspeaker, and the startle responses (RS3) were always directed away from it. This observation may suggest that the Eurasian otter has directional underwater hearing abilities. Another explanation could be that they learned to associate the speaker with noise, and that the directional displacement response was caused by visual cues instead of acoustic ones (e.g., observing the direction to the feeder).

The extrapolation of our study findings to wild otter populations introduces a notable degree of uncertainty, particularly due to the unique circumstances of the two individuals used in our experiments. These otters have been in human care and on public display since their early pup stages, experiencing continuous exposure to human presence and associated noise within the controlled exhibit environment. As a result, their reactions to acoustic stimuli may not necessarily mirror those of otters in the wild, being more habituated to anthropogenic sounds.

For otters to receive similar sound levels in the wild, the source intensity must be increased as the range to the source will be larger than in the experiments made here. At ranges between tens to hundreds of meters from the source, the source intensity (measured at 1 m) must be increased by at least 20–40 dB, depending on local sound propagation features. Spreading and absorption loss may both be higher and lower than what can be predicted from standard acoustic propagation model, so in situ measurements of sound spreading may be needed. In addition, background noise levels may be either higher or lower than the levels measured in the otter setup used for experiments here.

The use of controlled sound exposures demonstrate that otters are acoustically vigilant and respond behaviourally to sound. The animals showed strong responses to relatively weak signals of both 1 kHz and 14 kHz signals, and their behavioural responses were significantly related to sound level, but not to sound frequency. Implementing AHDs at 1 kHz to deter seals would therefore not solve the problem of not affecting the otters in their natural habitat, as suggested to be the case for the harbour porpoise^17^. Comparing the in–air hearing with other animals, it appears that the Eurasian otter has inferior low frequency hearing^15^ compared to other mustelids, such as the sea otter^16^, least weasel *(Mustela nivalis*)^35^, ferret (*Mustela putorius*)^36^, as well as pinnipeds, such as the harbour seal and northern fur seal (*Callorhinus ursinus*)^37,38^. As the otter forages under water and spends a large proportion of life under water, it is reasonable to assume that their hearing is adapted to this environment as indicated by our data.

The findings here will allow managers to make more informed decisions by incorporating species-specific information into risk assessments. We conclude that an acoustic harassment device has a high potential of affecting the diving and foraging success of otters, as shown for both individuals with a significant decrease in dive duration and increase in the time necessary to get fish out of the feeder. The highest received sound level used during these experiments was 145 dB re 1 µPa. In comparison, effective commercial AHDs/seal scarers have a sound level of 190–200 dB re 1 µPa^39–44^. Using commercially available AHD devices in rivers will obviously incur much stronger reactions from otters, probably including displacement and habitat exclusion by restricting access to the sea and movements between rivers. The behavioural and dive responses at 1 kHz were significant and similar to the 14 kHz signals and can therefore not be recommended as an alternative. Research concerning sound reception in Eurasian otters is still needed to measure specific underwater hearing sensitivity (audiograms). In order to better describe the acoustic biology of this species, more comparative studies are needed of auditory anatomy, neurophysiology and detailed characteristics of acoustic communication in their natural environment.

## Conclusion

The otters in this study showed clear behavioural response to both 1 and 14 kHz underwater sounds at 105–145 dB re 1 µPa. Otter sensitivity to sounds should be taken into consideration before installing AHDs to reduce presence of seals in areas with breeding and foraging otters or Natura 2000 areas where otters are under protection.

### Supplementary Information

Supplementary Information 1.

Supplementary Information 2.

Supplementary Information 3.

Supplementary Information 4.

## Data Availability

Data is available in supplementary files.
