# Supplementary material for: Response of Eurasian otters (Lutra lutra) to underwater acoustic harassment device sounds
Source: Sci Rep. 2024 Feb 29;14:4988. doi: 10.1038/s41598-024-55481-z (PMC10904746; doi:10.1038/s41598-024-55481-z)
Supplement: Supplementary file 1 — Supplementary Information 1. [file 41598_2024_55481_MOESM1_ESM.docx]

**APPENDIX**

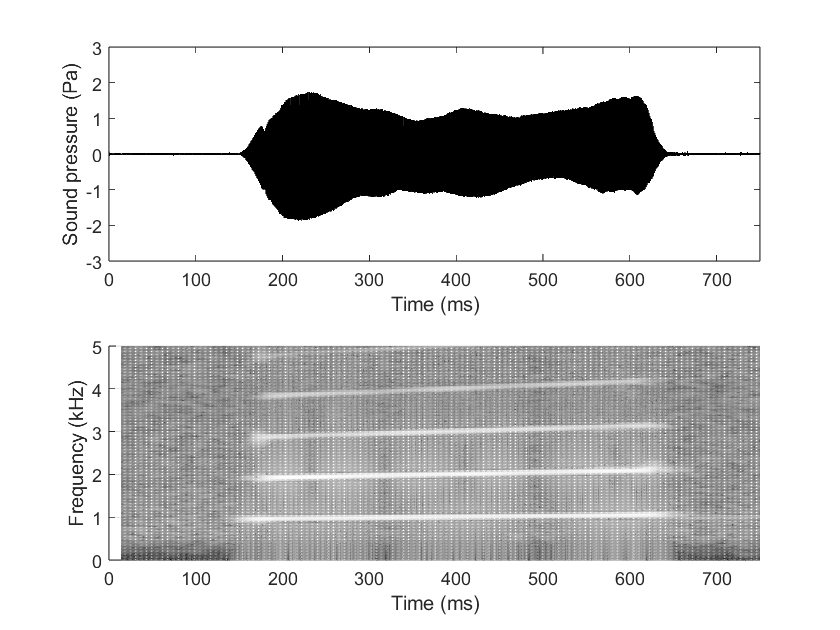

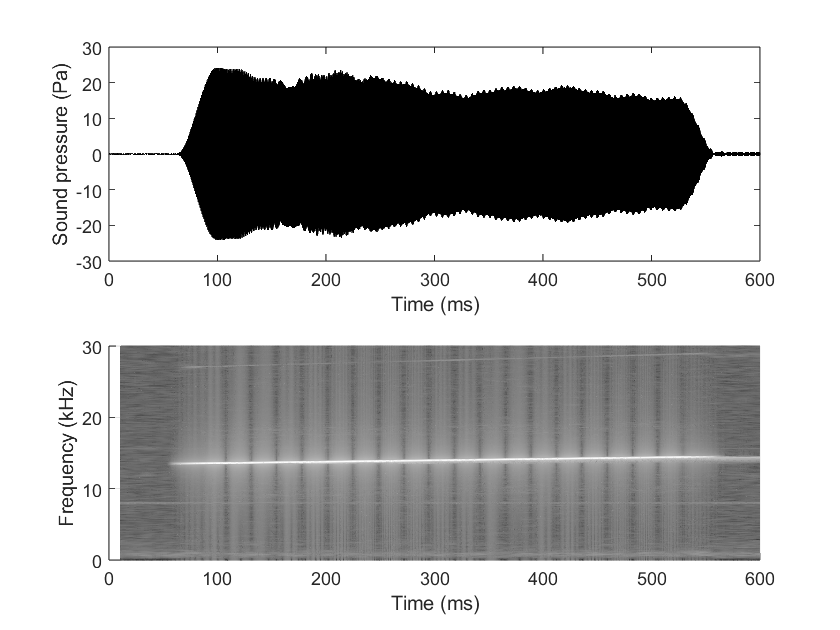


**Figure A1 *Top:*** Spectral noise levels in different locations of the otter pool between trials (black and green lines with circulation pump switched on and off, respectively, and stippled black line self-noise of recording equipment). Blue lines are minimum and maximum spectral noise levels at the location of playback with pump off. Welsh averaging, Hanning window, filtering bandwidth 46 Hz. ***Bottom****:* Oscillogram and spectrogram of stimulus recorded at 1 kHz (left) and 14 kHz (right) stimulus (16 bits, sampling rate 96 kHz). The spectrograms of the 1 and 14 kHz stimuli were made with an analysis bandwidth of 23 and 187 Hz, respectively, FFT size 2048, 95% overlap and Hanning window.
